# Prognostic value of elevated cardiac troponin I in patients with intracerebral hemorrhage

**DOI:** 10.1002/clc.23320

**Published:** 2019-12-18

**Authors:** Yangchun He, Qigong Liu, Jing Wang, Dao Wen Wang, Hu Ding, Wei Wang

**Affiliations:** ^1^ Division of Cardiology, Department of Internal Medicine, Tongji Hospital, Tongji Medical College Huazhong University of Science and Technology Wuhan China; ^2^ Hubei Key Laboratory of Genetics and Molecular Mechanism of Cardiological Disorders, Tongji Hospital, Tongji Medical College Huazhong University of Science and Technology Wuhan China; ^3^ Department of Neurology, Tongji Hospital, Tongji Medical College Huazhong University of Science and Technology Wuhan China

**Keywords:** cardiac troponin I, intracerebral hemorrhage, mortality, poor outcome, prognostic marker

## Abstract

**Background:**

Although cardiac troponin has been well established as diagnostic and prognostic makers for acute coronary heart disease, the prognostic value of elevated cardiac troponin in patients with intracerebral hemorrhage (ICH) was inconsistent and not systematically evaluated.

**Hypothesis:**

We proposed the hypothesis that the practical utility of cardiac troponin levels for prediction of mortality and poor outcome in ICH patients.

**Methods:**

A total of 1004 patients with ICH were retrospectively reviewed and qualified for further analysis from June 2012 to December 2015. The patients were divided into different groups based on measurements of cardiac troponin I (cTnI) at the time of admission and the following day. Multivariate Cox proportional hazards analysis were performed to determine the independent prognostic value of the cTnI for patients in‐hospital mortality and poor outcomes, the receiver operator characteristic (ROC) analysis was performed to assess the predictive value of cTnI, ICH score, and combination of them.

**Results:**

Serum cTnI level was an independent predictor in‐hospital mortality (positive vs negative, HR (hazard ratios) = 3.44, 95% CI (confidence interval) 1.66‐7.13, *P* < .001) and poor outcomes in patients with ICH (positive vs negative, HR = 6.69, 95% CI 4.25‐10.52, *P* < .001). Addition of cTnI to ICH score significantly improved the prognostic discrimination for both in‐hospital mortality and poor outcomes.

**Conclusion:**

Serum cTnI levels may be valuable as predictor for in hospital mortality and poor outcomes and may be useful in the risk stratification of ICH during hospitalization.

## 1 INTRODUCTION

Over the past three decades, stroke burden has emerged as a public health problem of epidemic proportions in China.^1^ Clinical studies have shown that the occurrence of intracerebral hemorrhage (ICH) in Chinese was remarkably frequenter that in Caucasians or developed countries.^2^, ^3^ Despite advance in general medical and critical care, ICH remained as the most severe form of stroke with high mortality rates (range from 22.8% to 27.5% for different race) and poor outcome of survivors.^4^, ^5^ Some inflammatory biological markers, including d‐dimer level^6^ and neutrophil‐to‐lymphocyte ratio,^7^ have shown potential relevance with prognostic in patients with ICH, while they were not sufficiently validated and routinely used in clinical practice. In addition to those inflammatory markers, the ICH score has been established and commonly used for estimating the risk of short‐term mortality or poor outcome of ICH patients from actual clinical data.^8^, ^9^, ^10^, ^11^ Given that biological markers involving in processes of ICH may add early prognostic information and be suitable targets for therapeutic research, other novel maker predisposing to ICH and its clinical sequel warrant further consideration.

Cardiac troponins, including cardiac troponin T and I, have been routinely used as sensitive and specific makers to diagnose acute coronary syndromes or myocardial damage such as acute myocarditis.^12^ In the setting of these syndromes, elevation of cardiac troponins also served as prognostic markers to predict adverse consequences of heart failure even in the absence of coronary artery stenosis.^13^ Moreover, cardiac troponin elevation has been reported in a number of nonmyocardial ischemic conditions including pulmonary embolism, sepsis syndrome, renal insufficiency and subarachnoid hemorrhage (SAH), and served as prognostic makers with different clinical implications.^13^, ^14^, ^15^ Particularly, elevated cardiac troponin is associated with an increasingly delayed cerebral ischemia, poor outcomes and death in SAH patients,^14^ playing an important role in predicting the prognostic value of the other type of hemorrhagic stroke.

Recently, elevation of cardiac troponin has been reported to occur in ICH patients along with only 1.2% of them died of cardiac causes.^16^ Although previous studies have been tried to address hypothesis that elevated cardiac troponin might serve as prognostic markers for prediction of adverse clinical events, the results were largely inconsistent and inconclusive with regard to ICH. The previous study demonstrated that elevated troponin levels were associated with higher mortality following ICH.^16^ Subsequently, the utility of elevated troponin levels for prediction of mortality was confirmed in surgical ICH patients,^17^ but was not found to be consistently associated with in‐hospital mortality in Chinese ICH patients.^18^ Small sample sizes (less than 240 stroke patients) and ethnic variability probably contribute to the negative results and discrepancies. This has prompted further efforts to determine whether cardiac troponin could provide valuable clinical prediction information for mortality as well as poor outcome in ICH patients with a relatively large cohort. We also confirmed the addition of cTnI to ICH score could significantly improve the prognostic discrimination for both mortality and poor outcome.

## 2 MATERIALS AND METHODS

### 2.1 Study subjects

This retrospective study included all patients with first‐ever primary ICH admitted directly to the Tongji hospital, from June 2012 to December 2015. All the subjects were identified by a systematic search in the clinical database (Haitai, Wuhan, China). Patients with ICH were diagnosed based on the strict neurological examination and results of cranial computed tomography (CT) or magnetic resonance Imaging (MRI) according to the International Classification of Diseases (*ninth Revision, codes 430‐438*). Patients diagnosed with primary SAH and ICH due to a structural cause (arteriovenous malformation, aneurysm, cavernous angioma, venous angioma, dural fistula) were excluded from present study. In addition, hemorrhages associated with trauma, brain tumor, hemorrhagic cerebral infarction, or thrombolytic treatment of ischemic stroke during hospitalization did not meet included criteria. Initially, a total of 1319 potential cases of hemorrhagic stroke were abstracted, but 61 were removed from analysis because they had at least one exclusion criterion: trauma (n = 6), hemorrhagic transformation with infarction (n = 25), hemorrhage into tumor (n = 11), subdural hematoma (n = 7), age < 18 at the time of event (n = 12). Finally, a total of 1004 patients with a diagnosis of ICH in Tongji Hospital (Hubei province, China) were finally enrolled in the retrospective medical records review (Figure S1). To determine whether these included patients were representative of all verified primary ICH patients and potential selection biases within the study, we compared the baseline characteristics and risk factors in original total sample (total group) and sub‐sample after the exclusion (included group). As shown in Table S1, the proportion of hyperlipidemia accounted for lower proportion in total patients vs included subjects (31.40% vs 38.25%, *P* < .001). No significant difference of the other baseline characteristics and risk factors was found between the total and included group.

This study was approved by the institutional review board of Tongji Hospital (Wuhan, China) and the data protection authorities. Owing to no breach of privacy and interference with clinical decisions related to patient care, informed consent was not required. All our present medical research was conducted according to the principles expressed in the Declaration of Helsinki.

### 2.2 Data collection and definition of risk factors

We obtained clinical data and background information, including history of hypertension, diabetes, hyperlipidemia, cerebrovascular disease (ischemic stroke or heart disease), smoking, and drinks. Similarly, these risk factors were define as previously reported.^19^

### 2.3 Cardiac testing

Serum concentrations of cTnI were measured by using the Abbott‐Architect Troponin I assay with the use of the Architect system (Abbott Diagnostics). According to the manufacturer and validation,^20^ the limit of detection of this assay was 0.010 ng/mL, the 99th‐percentile cutoff value was 0.028 ng/mL and the assay precision was 0.032 ng/mL, which achieved a coefficient of variation of less than 10%. An elevated cTnI was defined as plasma level greater than cutoff value of cTnI. Patients tested positive (cTnI ≥0.028 ng/mL) were divided into the dynamic group and stable group based on the dynamic changes of cTnI according to the previous studies.^21^, ^22^ Dynamic group was defined as a rise or fall of >30% with at least one cTnI value above the cutoff value, while the stable group was defined as cTnI with no significant dynamic changes.

When the patient's cTnI test is positive, a repeated measurement of cTnI was performed within 6 hours and a standard 12‐lead ECGs was routinely recorded. All the obtained ECG results of patient were collected, during which the changes of rhythm, heart rate, PR interval, QRS duration, QTc interval, and ST segment/T‐wave were analyzed. All intervals were determined using commercial ECG analysis software (12‐Lead Algorithm, GE Medical Systems, Connecticut). In addition, ECGs were employed as imaging evidence of new loss of viable myocardium or new wall motion abnormality by using a Vivid 7 ultrasound machine (GE Medical Systems).

### 2.4 Hematoma volume measurement

In order to assess the volume of intracranial hematomas, ABC/2 technique was used based on the initial CT or MRI in ICH subjects as previously description,^19^ where A referred to the maximum linear length in centimeter (cm), B related to the maximum width in cm, and C indicated the maximum depth in cm, respectively. The depth C was calculated by the number of slice where hematoma was visible multiplying by the slice thickness labeled in CT or MRI scan.^23^

### 2.5 Neurological scores assessment

Initially, brain injury of patients with ICH was assessed on admission using the Glasgow Coma Scale (GCS) score.^24^ As previously description, the ICH score was calculated by comprising information, such as age, GCS score, ICH volume, intraventricular involvement, and supratentorial vs infratentorial origin.^25^ While the clinical outcome was measured by modified Rankin Scale (mRS) scores at ICH day 14 (or discharge if earlier).^26^

### 2.6 In‐hospital complications and poor outcome assessment

In present study, the in‐hospital complications including abnormal ECG, left ventricle (LV) wall motion abnormality, hypotension, pulmonary edema, upper gastrointestinal hemorrhage, and acute renal insufficiency were recorded according to prespecified criteria based on the laboratory, imaging data as well as clinical manifestation. Abnormal ECG and LV wall motion abnormality were diagnosed by standard 12‐lead ECGs and echocardiograms, respectively. Hypotension was defined as a MAP (mean arterial pressure) <90 mmHg. Pulmonary edema was defined as the development of at least of two clinical findings as follow: rales, hypoxemia (PO_2_/FiO_2_ < 300) and pulmonary infiltrates on chest radiography. Upper gastrointestinal hemorrhage was defined as bleeding arising from the esophagus, stomach, or duodenum with observation of blood in vomit (hematemesis) or altered form in the stool (melena). As introduced by the kidney disease improving global outcomes (KDIGO) acute kidney injury work group,^27^ acute renal insufficiency was diagnosed if any one of the following was present: (a) increase in serum creatinine by ≥0.3 mg/dL (≥26.5 μmoL/L) within 48 hours; (b) increase in serum creatinine to ≥1.5 times baseline, which has occurred within the prior 7 days; (c) urine volume < 0.5 mL/kg/h for 6 hours. Clinical poor outcome was defined as severe functional disability (dependency, mRS score 3‐6) or mortality (death, mRS score 6).

### 2.7 Statistical analysis

Continuous variables were presented by employing mean values, SD (SD) and interquartile range (IQR) to reflect median values, normally distributed data and nonparametric data, respectively. The distributions of continuous variables were tested for normality by use of normality Q‐Q plots and 1‐sample Kolmogorov‐Smirnov test. Continuous variables were compared by means of *t* test and analysis of variance for normally distributed data, and nonparametric Mann‐Whitney *U* test for abnormally distributed data (cTnI, GCS, ICH, and mRS scores). Spearman's correlation analysis was performed to test the relationships between cTnI and neurological scores (GCS, ICH, and mRS scores). Categorical variables were presented in absolute values and percentages and were compared by the Pearson *χ* ^2^ or Fisher exact test, where appropriate.

Multivariate Cox proportional hazards analysis were used to assess the independent prognostic value of the cTnI for in‐hospital short‐term (30 days) mortality and poor outcomes with adjustment of covariates. Three independent multivariable Cox models were constructed with these cTnI entered as a continuous variable (logarithmically transformed because of a skewed distribution) or a dichotomous variable (negative vs positive) and as three categorical variables (negative vs stable and negative vs dynamic). Cox regression plots of the survival curves were generated according to cTnI levels and cumulative rates of survival between different groups using the log‐rank test.

Receiver operator characteristic (ROC) analysis was performed to determine the predictive value of cTnI, ICH score, and their combination value for mortality and poor outcomes. The optimal cutoff value was determined by maximal Youden index with sensitivity and specificity.^28^ The comparisons between the areas under the ROC curves (AUC) were performed as a recommended method by DeLong et al.^29^

Statistical analysis was performed with spss 22.0 (spssInc., Chicago, Illinois) and MedCalc 19.2 Statistical Software (MedCalc software, Mariakerke, Belgium) for Windows (MicrosoftCorp, Redmond, Washington). Two‐sided test was performed during whole experiment, and *P* values less than .05 were thought to reach statistical significance.

## 3 RESULTS

### 3.1 Distribution of serum cTnI levels

Among 1004 patients with ICH, a total of 275 patients had serum cTnI level above the reference (≥0.028 ng/mL, 27.4%). As shown in Figure S2, the distributions of serum cTnI level deviated from a strict normal distribution, and median serum cTnI level in these patients was 0.010 ng/mL.

### 3.2 Clinical characteristics of patients with ICH

Baseline characteristics and risk factors of patients with ICH according to serum cTnI concentration were presented in Table 1. Initially, we divided all the patients into two groups by cTnI reference value: a cTnI negative group (cTnI < 0.028 ng/mL, n = 729) and a positive group (≥0.028 ng/mL, n = 275). Positive group were further divided into two groups (constant, n = 187 and dynamic, n = 88) according to the elevation of cTnI. Compared with cTnI negative group patients, dynamic group has higher systolic blood pressure (*P* < .05), the percentage of history of heart disease and anti‐coagulation (all the *P* < .01). However, there were no significant differences for other documented risk factors between the cTnI negative group and constant group as well as dynamic group. As expected, the admission characteristics such as ICH volume (>30 mL), GCS, ICH, and mRS scores were significantly different between the cTnI negative group with constant or dynamic groups (all the *P* value < .001, Table S2).

**Table 1:** Baseline characteristics and risk factors in ICH patients^a^

		cTnI positive (≥0.028 ng/mL) n = 275	
	cTnI negative (<0.028 ng/mL)	cTnI stable	cTnI dynamic
Characteristics	n = 729	n = 187	n = 88
Age (years)	55.13 ± 11.38	56.45 ± 13.44	56.41 ± 14.33
Male, n (%)	458 (62.83)	126 (67.38)	54 (61.36)
SBP (mmHg)	154.60 ± 22.57	158.35 ± 26.90*	158.90 ± 26.91^#^
DBP (mmHg)	91.75 ± 15.97	94.25 ± 19.01	91.75 ± 18.75
Hypertension, n (%)	428(58.71)	115 (61.50)	53 (60.23)
Diabetes, n (%)	51 (7.00)	10 (5.35)	5 (5.68)
Hyperlipidemia, n (%)	284 (38.96)	71 (37.97)	29 (32.95)
Ischemic stroke, n (%)	88 (12.07)	27 (14.44)	12 (13.64)
Heart disease,^b^ n (%)	29 (3.98)	13 (6.95)	13 (14.77)*
Smokers (current or former), n (%)	157(21.54)	41 (21.93)	23(26.14)
Drinkers, n (%)	158 (21.67)	42 (22.46)	19 (21.59)
Anti‐coagulation,^c^ n (%)	8(1.10)	2 (1.07)	6 (6.82)*

*Note*: Test for differences between cTnI negative patients and subgroups of cTnI positive patients, *P < .01 and ^#^ *P* < .05.

Abbreviations: cTnI, cardiac troponin I; DBP, diastolic blood pressure; ICH, intracerebral hemorrhage; n, number of individuals; SBP, systolic blood pressure.

a Categorical variables are presented in absolute values with percentages, n (%); Continuous variables are presented as mean (±SD).

b History of myocardial infarction, atrial fibrillation, prosthetic valve, cardiac bypass, cardiac angioplasty, or pacemaker.

c Warfarin, heparin, or low‐molecular‐weight heparin.

### 3.3 In‐hospital complications and clinical poor outcome

**Figure 1:**
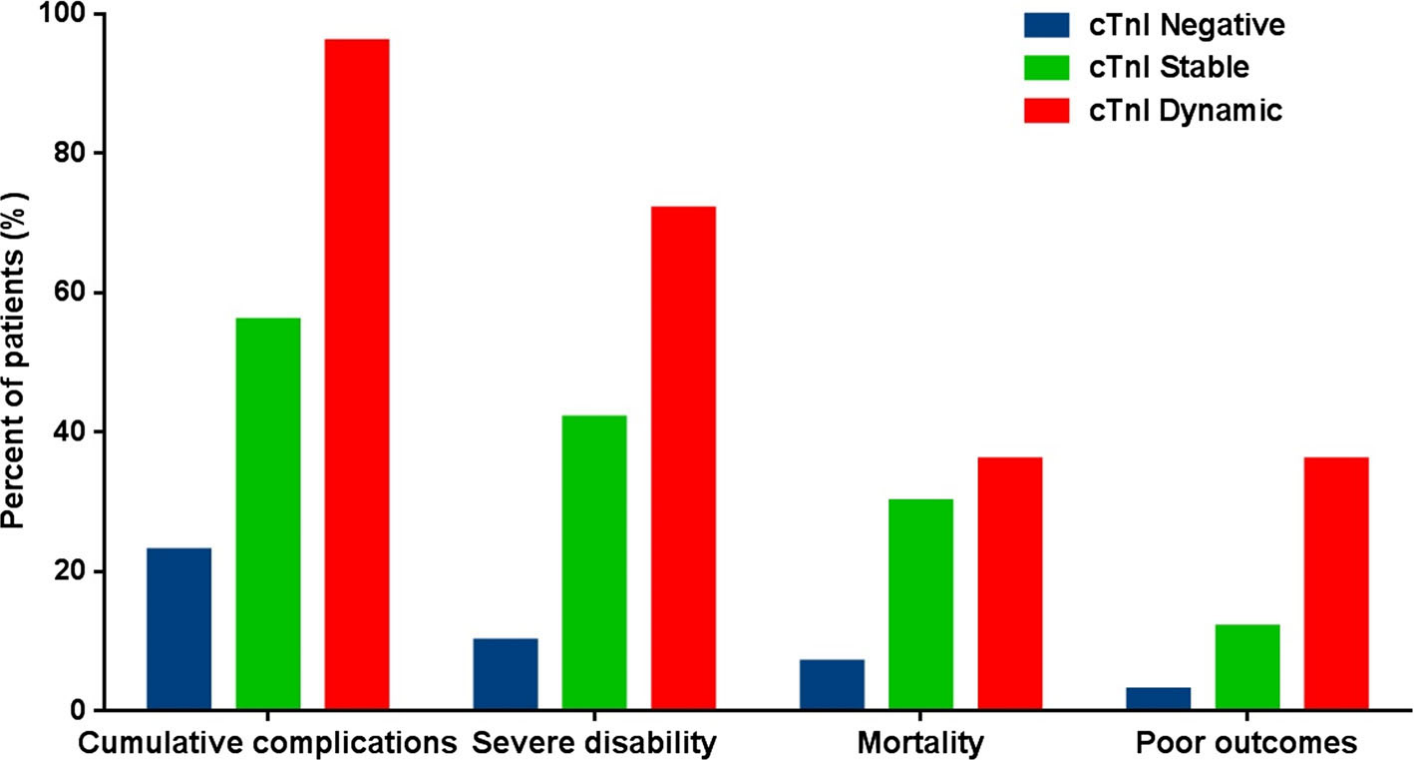
Prevalence of cumulative in‐hospital complications, severe disability, mortality, and poor outcomes according to cTnI levels

As shown in Figure 1, compared with cTnI negative group patients (constant, dynamic vs negative), patients with cTnI level above the reference (both constant and dynamic groups) had higher cumulative in‐hospital complications (56.68%, 96.59% vs 23.18%, both *P* < .01) and clinical poor outcomes (42.78%, 72.72% vs 10.15%, both *P* < .01), including severe disability (30.48%, 36.36% vs 7.13%, both *P* < .01) and mortality (12.30%, 36.36% vs 3.02%, both *P* < .01).

### 3.4 Correlation analysis between cTnI with GCS, ICH and mRSscores

Subsequently, we performed correlation analysis between serum peak cTnI levels and neurological scores. As shown in Table S3, cTnI levels had positive correlations with ICH score (r = .330, *P* < .001) and mRS score (r = .350, *P* < .001), in contrast, had negative correlations with GCS score (r = −.296, *P* < .001).

### 3.5 Prognostic value of cTnI for prediction of in‐hospital short‐term mortality of ICH patients

**Figure 2:**
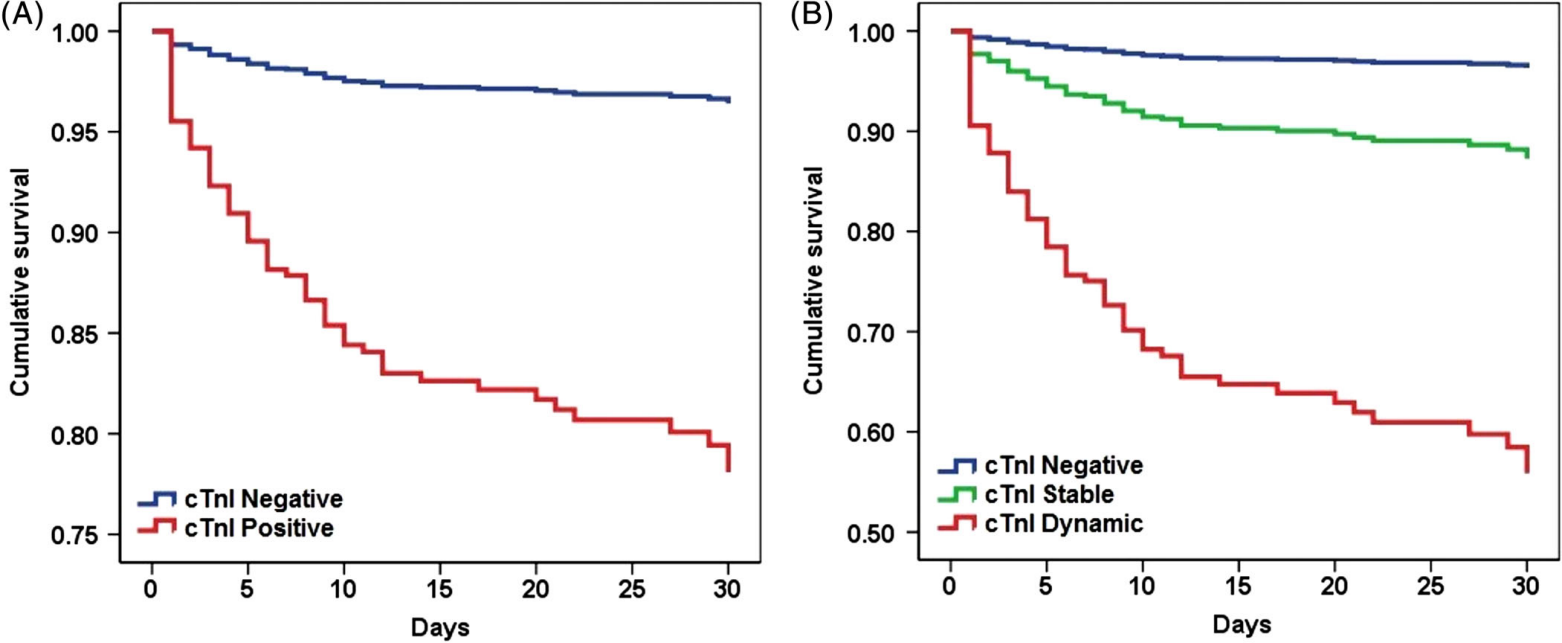
Cox regression plots of the survival curves reflecting cumulative proportion of survivals of patients with intracerebral hemorrhage according to cTnI levels

A total of 77 patients (7.7%) died during a median hospital stay of 5 days (IQR range from 2 to 12 days), among whom 61 patients (79.2%) died directly due to ICH. Baseline characteristics according to survival status were summarized in Table S4. As shown in Table 2, serum cTnI level was an independent predictor for 30 days mortality when it was considered either as two categories by the reference value (positive vs negative, HR = 3.44, CI 1.66‐7.13, *P* < .001) or as three categories by the elevation of cTnI (constant vs negative HR = 2.79, CI 1.31‐5.98, *P* < .001; dynamic vs negative HR = 10.59, CI 4.76‐23.55, *P* < .001), but not as a continuous variable (HR = 1.21, CI 0.73‐1.98, *P* = .471). In addition, Cox regression analysis showed that in cumulative 30 days mortality was significantly higher in patients with positive cTnI compared with negative cTnI (Log rank *P* < .001, Figure 2A). When further elevated cTnI, the 30 days mortality significantly increased along with elevation of cTnI (Log rank *P* < .001, Figure 2B).

**Table 2:** Multivariate Cox regression for predictors of in‐hospital short‐term mortality and poor outcomes of ICH patients

Variables	HRs (95% CI)	*P* value
**Mortality**		
Model 1		
cTnI continuous	1.21 (0.73 to 1.98)	.471
Model 2		
cTnI negative	Reference	
cTnI positive	3.44 (1.66 to 7.13)	<.001
Model 3		
cTnI negative	Reference	
cTnI stable	2.79 (1.31 to 5.98)	<.001
cTnI dynamic	10.59 (4.76 to 23.55)	<.001
**Poor outcomes**		
Model 1		
hs‐cTnI continuous	1.74 (1.24 to 2.46)	.001
Model 2		
hs‐cTnI negative	Reference	
hs‐cTnI positive	6.69 (4.25 to 10.52)	<.001
Model 3		
hs‐cTnI negative	Reference	
hs‐cTnI stable	5.47 (3.46 to 8.64)	<.001
hs‐cTnI dynamic	14.40 (7.69 to 29.94)	<.001

*Note*: Model 1, cTnI as a continuous variable (logarithmically transformed); Model 2, cTnI as a dichotomous variable (negative vs positive); Model 3, cTnI as three categorical variables (negative vs stable and negative vs dynamic). Multivariable adjusted for age, gender, history of hypertension, diabetes, hyperlipidemia, ischemic stroke, heart disease, smoking status, drinking status anti‐coagulation medication, admission characteristics (including whether ICH volume > 30 mL, GCS score, ICH score, mRS score) and in‐hospital complications; Poor outcomes were defined as severe disability and mortality.

Abbreviations: CI, confidence interval; GCS, Glasgow Coma Scale; HRs, hazard ratios; ICH, intracerebral hemorrhage; mRS, modified Rankin Scale.

### 3.6 Prognostic value of cTnI for predictors for prediction of poor outcomes of ICH patients

In total, clinical poor outcomes were observed in 217 patients (2.2%). Table S5 illustrated the baseline characteristics according to with/without poor outcomes. As shown in Table 2, serum cTnI level was independently associated with poor outcomes regardless of being considered as a continuous variable (HR = 1.74, CI 1.24‐2.46, *P* = .001), two categories by the reference value (positive vs negative, HR = 6.69, CI 4.25‐10.52, *P* < .001) and three categories by the elevation of cTnI (constant vs negative, HR = 5.47, CI 3.46‐8.64, *P* < .001; dynamic vs negative, HR = 14.40, CI 7.69‐29.94, *P* < .001).

### 3.7 Adding cTnI to ICH score improves the predicting value of in‐hospital short‐term mortality and poor outcome in ICH patients

**Figure 3:**
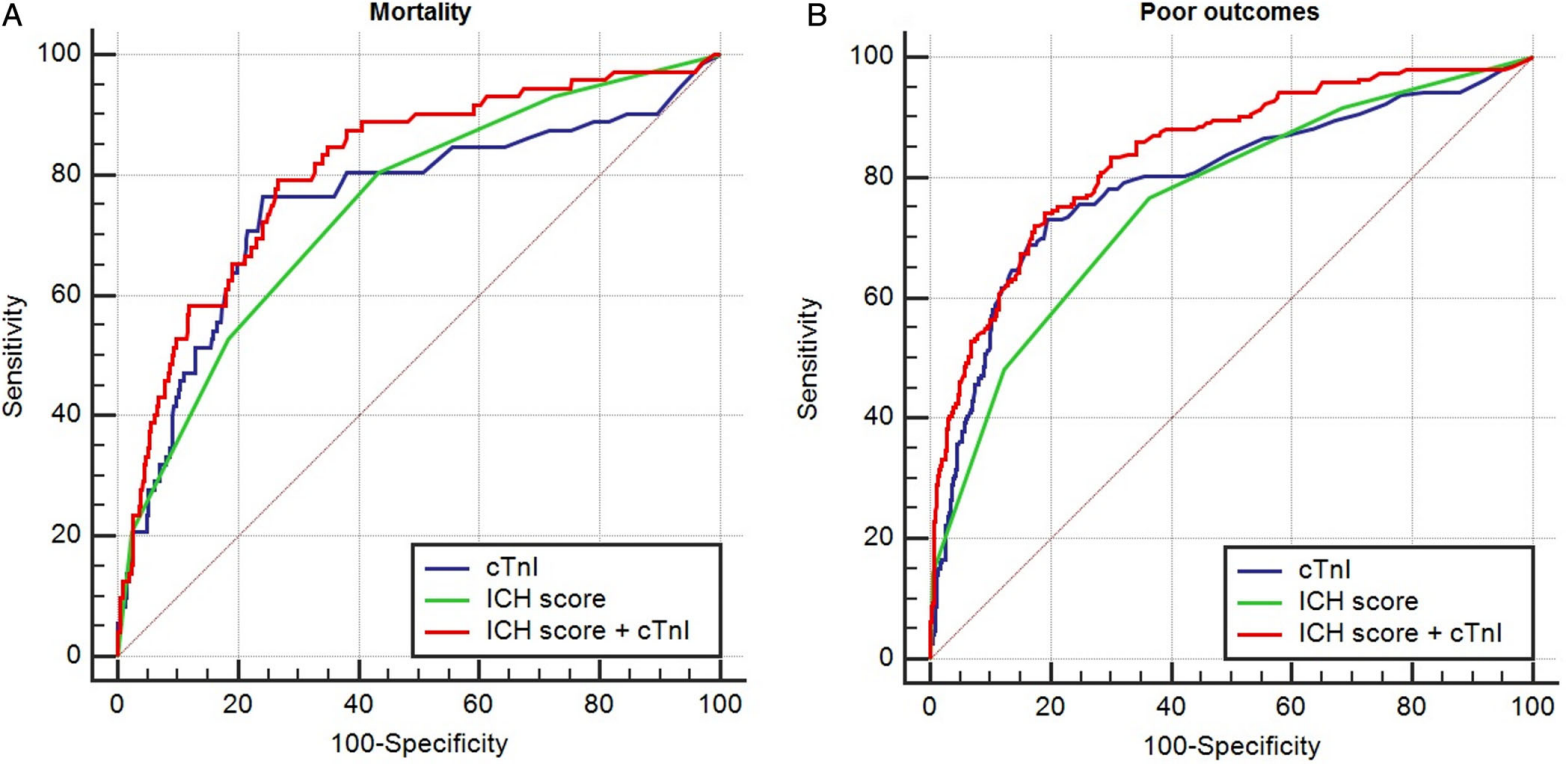
. Receiver operator characteristic (ROC) curves for in‐hospital mortality and poor outcomes. Area under receiver operating characteristic (AUC) curve values for using cTnI, ICH score and combination of them in predicting in‐hospital short‐term (30‐days) mortality (A) and poor outcomes (B) of patients with ICH

As shown in Table S6, the diagnostic accuracy of cTnI, ICH score, and combined of them in predicting of in‐hospital short‐term mortality and poor outcomes of ICH patients was moderate. In particular, the addition of cTnI to ICH score significantly improved the prognostic discrimination for both mortality and poor outcomes (both *P* < .001, Figure 3 and Table S7).

## 4 DISCUSSION

In present study, we examined the utility of cTnI as prognostic makers in patients with ICH. The main findings of this study can be summarized as follows: (a) serum peak cTnI levels was correlated with GCS, ICH and mRS scores in patients with ICH; (b) serum cTnI elevated in ICH patients and along with high proportion of in‐hospital complications and adverse events; (c) elevation of cTnI, especially dynamic change of cTnI, were independently associated with incidence of in‐hospital mortality and poor outcomes; (d) adding cTnI to ICH score further increased the prognostic discrimination for both in‐hospital mortality and poor outcome of ICH patients. Thus, cTnI seems to be useful as an additive monitoring marker in patients with ICH.

The mechanisms of elevated cTnI in some of the patients with ICH was complicated and incompletely understood. Cardiac troponins are considered the most sensitive and specific biochemical markers for cardiac injury.^30^ Thus, elevation and dynamic changes of serum cTnI levels are indicators of cardiac injury after ICH. The excess of catecholamines was found in patients with ICH, which peaked on the first week and then declined.^31^ Given that catecholamine was capable of producing myocardial necrosis, even in the nonischemic heart,^32^ the catecholamine surge theory has been reasonable linked to cardiac injury after ICH.^33^ In addition, systemic inflammatory responses are activated after ICH and might contribute to myocyte injury and cell death.^34^ Several other putative causes were also responsible for the observed elevated cTnI levels, including cardiopulmmonary disease, renal insufficiency, and ICH patients with neurological deterioration.^35^ In present study, elevated cTnI was associated with high frequency of pulmonary edema and acute renal insufficiency.

Several important limitations of present study must be acknowledged. First, this was a single‐center, retrospective study. Thus, the main findings of our study still need multi‐centers and prospective studies to be further verified in order to assessment prognostic value of cTnI in patients with ICH. Second, whether adding troponin to the existing blood prognostic markers improves the predicting value of clinical outcome in ICH patients warrants further assessment. Third, the utility of cTnI for the identification of patients with ICH at high‐risk of long‐term mortality as well as adverse outcomes would greatly strengthen the importance but not determinate in present study. We consider that further long‐term follow‐up period, and many end‐point studies are mandatory for confirming the correlation between cTnI and the prognosis of ICH. Fourth, because serial measurements of cTnI were not available, we could not address the important issue that whether any appropriate treatment to reduce cTnI might help us to improve outcome in patients with ICH. Finally, the weakness of present study is that current commercial version of high‐sensitivity cardiac troponin I (hs‐cTnI) assay was not routinely used in our central lab (before the year of 2016). Given that the diagnostic threshold to the 99th percentile is lower in hs‐cTnI assay when compared with cTnI assay (0.014 ng/mL vs 0.028 ng/mL), the increased number of patients presenting with an elevated cTnI level that needs further assessment of the prognostic information.

In this study, we demonstrated that elevation of cTnI after ICH has important prognostic predictive value, particularly regarding dynamic changes in cTnI for prediction of in‐hospital mortality and poor outcomes. In the future, we need larger prospective cohort study to confirm these findings and test the utility of risk stratification based on cTnI, which could guide clinicians to implement appropriate therapeutic strategies.

## CONFLICT OF INTEREST

The authors declare no potential conflict of interests.

## Supporting information


**Appendix S1.** Supporting Information

**Figure S1.** Study flow diagram. SAH = Subarachnoid hemorrhage; ICH = intracerebral hemorrhage.

**Figure S2.** Distribution patterns of peak cTnI levels in ICH patients. Histogram showing the patterns of distribution of peak cardiac troponin I levels in 1004 patients with intracerebral hemorrhage (ICH).N indicates the number of individuals. The green line indicates the 99th percentile of the applied cTnI assay (0.028 ng/mL) in general population.
