# Supplementary material for: Prognostic value of elevated cardiac troponin I in patients with intracerebral hemorrhage
Source: Clin Cardiol. 2019 Dec 18;43(4):338–45. doi: 10.1002/clc.23320 (PMC7144484; doi:10.1002/clc.23320)
Supplement: Supplementary file 1 — Appendix S1. Supporting Information [file CLC-43-338-s001.docx]

| **Supplementary Table1. Comparison of baseline characteristics and risk factors in total and included ICH patients** | | | |
| --- | --- | --- | --- |
| **Characteristics** | **Total**  **(n=1258)** | **Include**  **(n=1004)** | ***p* value** |
| Age (years) | 55.15±11.95 | 55.54±12.10 | 0.435 |
| Male, n (%) | 820 (65.18) | 638 (63.55) | 0.419 |
| SBP (mmHg) | 153.67±24.36 | 155.67 ±23.87 | 0.050 |
| DBP (mmHg) | 91.23 ±17.04 | 92.22 ±16.84 | 0.719 |
| Hypertension, n (%) | 744 (59.14) | 596 (59.36) | 0.915 |
| Diabetes, n (%) | 84 (6.68) | 66 (6.57) | 0.922 |
| Hyperlipidemia, n (%) | 395 (31.40) | 384 (38.25) | 0.001 |
| Ischemic stroke, n (%) | 180 (14.31) | 127 (12.65) | 0.252 |
| Heart disease^b^, n (%) | 69 (5.48) | 55 (5.48) | 0.994 |
| Smokers (current or former), n (%) | 312 (24.80) | 221 (22.00) | 0.120 |
| Drinkers, n (%) | 293 (23.30) | 219 (21.71) | 0.404 |
| Anticoagulation^c^, n (%) | 18 (1.43) | 16 (1.59) | 0.862 |
| ICH, intracerebral hemorrhage; n, number of individuals; SBP, systolic blood pressure; DBP diastolic blood pressure; cTnI, cardiac troponin I. | | | |
| ^a^Categorical variables are presented in absolute values with percentages, n (%); Continuous variables are presented as mean (±SD); | | | |

^b^History of myocardial infarction, atrial fibrillation, prosthetic valve, cardiac bypass, cardiac angioplasty or pacemaker;

^c^Warfarin, heparin, or low-molecular-weight heparin.

| **Supplementary Table 2. Admission characteristics, in hospital complications and poor outcomes in ICH patients^a^** | | | |
| --- | --- | --- | --- |
| **Characteristics** | **cTnInegative**  **(****<0.028 ng/ml)** | **cTnI positive (≥0.028 ng/ml) n=275** | |
|  |  | **cTnI Stable** | **cTnI Dynamic** |
|  | **n=729** | **n=187** | **n=88** |
| Admission characteristics | | | |
| ICH volume> 30ml, n (%) | 224 (30.72) | 94 (50.27)* | 57 (64.77)* |
| GCS score , IQR | 13 (10, 15) | 11(8, 13)* | 10 (6, 12)* |
| ICH score , IQR | 1 (0, 2) | 2(1, 3)* | 2 (1, 3)* |
| mRS score, IQR | 1 (0, 2) | 2(1, 3)* | 2 (1, 3)* |
| In hospital complications | | | |
| Abnormal ECG , n (%) | 15 (2.01) | 5 (2.67) | 24 (27.27)* |
| LV wall motion abnormality, n (%) | 1 (0.14) | 1 (0.53) | 5 (5.68)* |
| Hypotension, n (%) | 2 (0.27) | 6 (3.21)* | 7 ( 7.95)* |
| Pulmonary edema, n (%) | 74 (10.15) | 47 (25.13)* | 22 (25.00)* |
| Upper gastrointestinal hemorrhage, n (%) | 47(6.45) | 12 (6.42) | 14 (15.91)* |
| Acute renal insufficiency, n (%) | 30(4.11) | 35 (18.72)* | 13 (14.77)* |
| Poor outcomes^b^ (30-days) | | | |
| Severe disability, n (%) | 52 (7.13) | 57 (30.48)* | 32 (36.36)* |
| Mortality (all cause), n (%) | 22 (3.02) | 23 (12.30)* | 32 (36.36)* |
| ICH, intracerebral hemorrhage; n, number of individuals; GCS, Glasgow Coma Scale; IQR, interquartile range; mRS, modified Rankin Scale; cTnI, cardiac troponin I;  Test for differences between cTnI negative patients and subgroups of cTnI positive patients, *p<0.01; | | | |
| ^a^Categorical variables are presented in absolute values with percentages, n (%); Continuous variables are presented as mean (±SD) or median with interquartile range; | | | |
| ^b^Poor outcomes were defined as severe disability and mortality of ICH patients. | | | |

| **Supplementary Table 3. Partial correlations between peak cTnI levels with GCS, ICH and mRS scores.** | | | | |
| --- | --- | --- | --- | --- |
|  | **cTnI^a^** | | | |
|  |  | **r** |  | ***p*** |
| **GCS score** |  | -0.296 |  | <0.001 |
| **ICHscore** |  | 0.330 |  | <0.001 |
| **mRS score** |  | 0.350 |  | <0.001 |
| cTnI, cardiac troponin I; GCS, Glasgow Coma Scale; mRS, modified Rankin Scale; r, correlation coefficient; p, p value.  ^a^ cTnI as a continuous variable (logarithmically transformed). | | | | |

| **Supplementary Table 4. Comparison of baseline characteristics and risk factors in survival and death patients with ICH^a^** |
| --- |

| **Characteristics** | **Survival (n=927)** | **Death (n=77)** | ***p* value** |
| --- | --- | --- | --- |
| Age (years) | 55.50±11.50 | 55.97 ±17.90 | 0.749 |
| Male, n (%) | 593 (63.97) | 45 (58.44) | 0.333 |
| SBP (mmHg) | 155.76 ±23.28 | 154.61 ± 30.25 | 0.272 |
| DBP (mmHg) | 92.53 ±16.45 | 88.52 ±20.76 | 0.061 |
| Hypertension, n (%) | 556 (59.98) | 40 (51.95) | 0.168 |
| Diabetes n, (%) | 60 (6.47) | 6 (7.79) | 0.653 |
| Hyperlipidemia, n (%) | 351 (37.86) | 33 (42.86) | 0.386 |
| Ischemic stroke, n (%) | 116 (12.51) | 11(14.29) | 0.653 |
| Heart disease^b^, n (%) | 47 (5.07) | 8 (10.39) | 0.049 |
| Smokers (current or former), n (%) | 206 (22.22) | 15 (19.48) | 0.577 |
| Drinkers, n (%) | 205 (22.11) | 14 (18.18) | 0.422 |
| Anticoagulation^c^, n (%) | 12 (1.29) | 4(5.19) | 0.031 |
| cTnI (ng/ml), IQR | 0.01 (0, 0.03) | 0.11 (0.02, 0.72) | <0.001 |
| cTnI negative , n (%) | 707 (76.27) | 22 (28.57) | NA |
| cTnI positive, n (%) | 220 (23.73) | 55 (71.43) | <0.001 |
| cTnI stable, n (%) | 164 (17.69) | 23 (29.87) | <0.001 |
| cTnI dynamic, n (%) | 56 (6.04) | 32 (41.56) | <0.001 |
| ICH, intracerebral hemorrhage; n, number of individuals; SBP, systolic blood pressure; DBP diastolic blood pressure; cTnI, cardiac troponin I; NA, not available. | | | |
| ^a^Values are expressed as percentages or mean ± SD; | | | |
| ^b^History of myocardial infarction, atrial fibrillation, prosthetic valve, cardiac bypass, cardiac angioplasty or pacemaker;  ^C^Warfarin, heparin, or low-molecular-weight heparin. | | | |

| **Supplementary Table 5. Comparison of baseline characteristics and risk factors in ICH patients with and without poor outcomes^a^** |
| --- |

| **Characteristics** | **Without poor outcomes**  **(n=787)** | **Poor outcomes**  **(n=217)** | ***P* value** |
| --- | --- | --- | --- |
| Age (years) | 54.98 ±11.36 | 57.60 ±14.34 | 0.002 |
| Male n, (%) | 508 (64.55) | 130 (59.91) | 0.209 |
| SBP (mmHg) | 155.33 ±22.94 | 156.91 ±27.00 | 0.221 |
| DBP (mmHg) | 92.59±16.15 | 90.86 ±19.09 | 0.779 |
| Hypertension n, (%) | 466 (59.21) | 130 (59.91) | 0.853 |
| Diabetes n, (%) | 51 (6.48) | 15 (6.91) | 0.820 |
| Hyperlipidemia n, (%) | 307(39.00) | 77 (35.48) | 0.344 |
| Ischemic stroke n, (%) | 98 (12.45) | 29(13.36) | 0.721 |
| Heart disease^b^ n, (%) | 34 (4.32) | 21 (9.68)* | 0.002 |
| Smokers (current or former) n, (%) | 168 (21.35) | 53 (24.42) | 0.333 |
| Drinkers n, (%) | 171 (21.73) | 48 (22.12) | 0.902 |
| Anticoagulation^c^n, (%) | 9 (1.14) | 7 (3.23) | 0.058 |
| cTnI (ng/ml), IQR | 0.01 (0.00, 0.02) | 0.08 (0.01, 0.32) | <0.001 |
| cTnI negative | 655 (83.23) | 74 (34.10) | NA |
| cTnI positive | 132 (16.77) | 143 (65.90) | <0.001 |
| cTnI stable | 108 (13.72) | 79 (36.41) | <0.001 |
| cTnI dynamic | 24 (3.05) | 64 (29.49) | <0.001 |
| ICH, intracerebral hemorrhage; n, number of individuals; IQR, interquartile range; Poor outcomes were defined as severe disability and mortality. | | | |
| ^a^Values are expressed as percentages or mean ± SD;  ^b^History of myocardial infarction, atrial fibrillation, prosthetic valve, cardiac bypass, cardiac angioplasty or pacemaker;  ^C^Warfarin, heparin, or low-molecular-weight heparin. | | | |

| **Supplementary Table 6. Diagnostic value of using cTnI , ICH and combined of them in predicting in hospital short-term (30-days) mortality and poor outcomes of patients with ICH.** | | | | | |
| --- | --- | --- | --- | --- | --- |
| **Variables** | **AUC (95%CI)^a^** | **Cut-off**  **value** | **Sens** | **Spec** | **Youden**  **index** |
| **Mortality** |  | | | |  |
| cTnI^b^ | 0.75 (0.72-0.78) | 0.092 | 0.58 | 0.83 | 0.41 |
| ICH score | 0.75 (0.71-0.78) | 2.5 | 0.53 | 0.82 | 0.34 |
| ICH score +cTnI | 0.81 (0.78-0.84) | NA | 0.78 | 0.74 | 0.52 |
| \| **Poor outcomes^c^** \| \|  \| \| \| \| \|  \| \| --- \| --- \| --- \| --- \| --- \| --- \| --- \| --- \| \| cTnI \| 0.79 (0.76-0.82) \| \| 0.034 \| 0.73 \| 0.80 \| 0.54 \| \| \| ICH score \| 0.76 (0.73-0.79) \| \| 2.5 \| 0.48 \| 0.88 \| 0.36 \| \| \| ICH score+cTnI \| 0.84 (0.81-0.87) \| \| NA \| 0.76 \| 0.81 \| 0.57 \| \|   cTnI, cardiac troponin I; ICH, intracerebral hemorrhage; AUC, area under the receiver operating characteristic curve; CI, confidence interval; Sens, sensitivity; Spec, specificity;  ^a^all the p values were less than 0.001;  ^b^cTnI as a continuous variable (logarithmically transformed);  ^c^Poor outcomes were defined as severe disability and mortality. | | | | | |

| **Supplementary Table 7. Pairwise comparison of ROC curves.** | | |  |  |
| --- | --- | --- | --- | --- |
| **Variables** | **Difference between areas (95% CI)** | ***p*value** | |  |
| **Mortality** |  | | |  |
| cTnI | +0.01 (0.08 to -0.08) | 0.903 | |  |
| ICH score + cTnI | +0.06 (0.02 to 0.11) | <0.001 | |  |
| ICH score | reference | NA | |  |
| \| **Poor outcomes** \| \|  \| \|  \| \| \| --- \| --- \| --- \| --- \| --- \| --- \| \| cTnI \| +0.03 (0.08 to -0.02) \| \| 0.203 \| \| \| ICH score + cTnI \| +0.08 (0.05 to 0.11) \| \| <0.001 \| \| \| ICH score \| reference \| \| NA \| \|   ROC, receiver operating characteristic; CI, confidence interval; cTnI, cardiac troponin I;  NA, not available. | | | | |

**Figure Legends**

**Supplementary Figure1.** Study flow diagram.

SAH = Subarachnoid hemorrhage; ICH = intracerebral hemorrhage.

**Supplementary Figure 2.** Distribution patterns of peak cTnI levels in ICH patients. Histogram showing the patterns of distribution of peak cardiac troponin I levels in 1004 patients with intracerebral hemorrhage (ICH).N indicates the number of individuals. The green line indicates the 99^th^ percentile of the applied cTnI assay (0.028 ng/ml) in general population.
