# Supplementary figures and images for: Prognostic value of elevated cardiac troponin I in patients with intracerebral hemorrhage
Source: Clin Cardiol. 2019 Dec 18;43(4):338–45. doi: 10.1002/clc.23320 (PMC7144484; doi:10.1002/clc.23320)

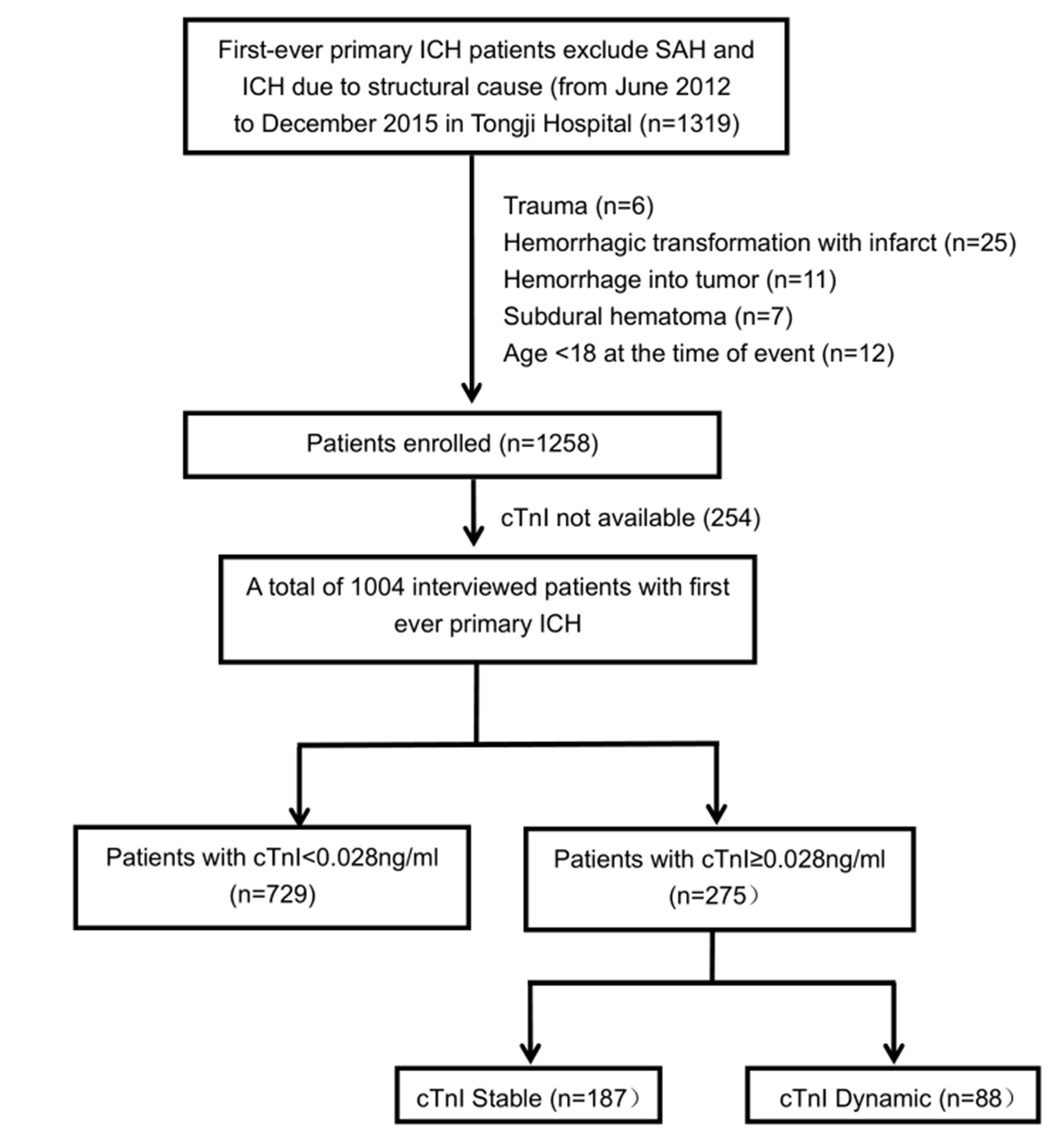

Supplement: Supplementary file 2 — Figure S1. Study flow diagram. SAH = Subarachnoid hemorrhage; ICH = intracerebral hemorrhage. [file CLC-43-338-s002.tif]

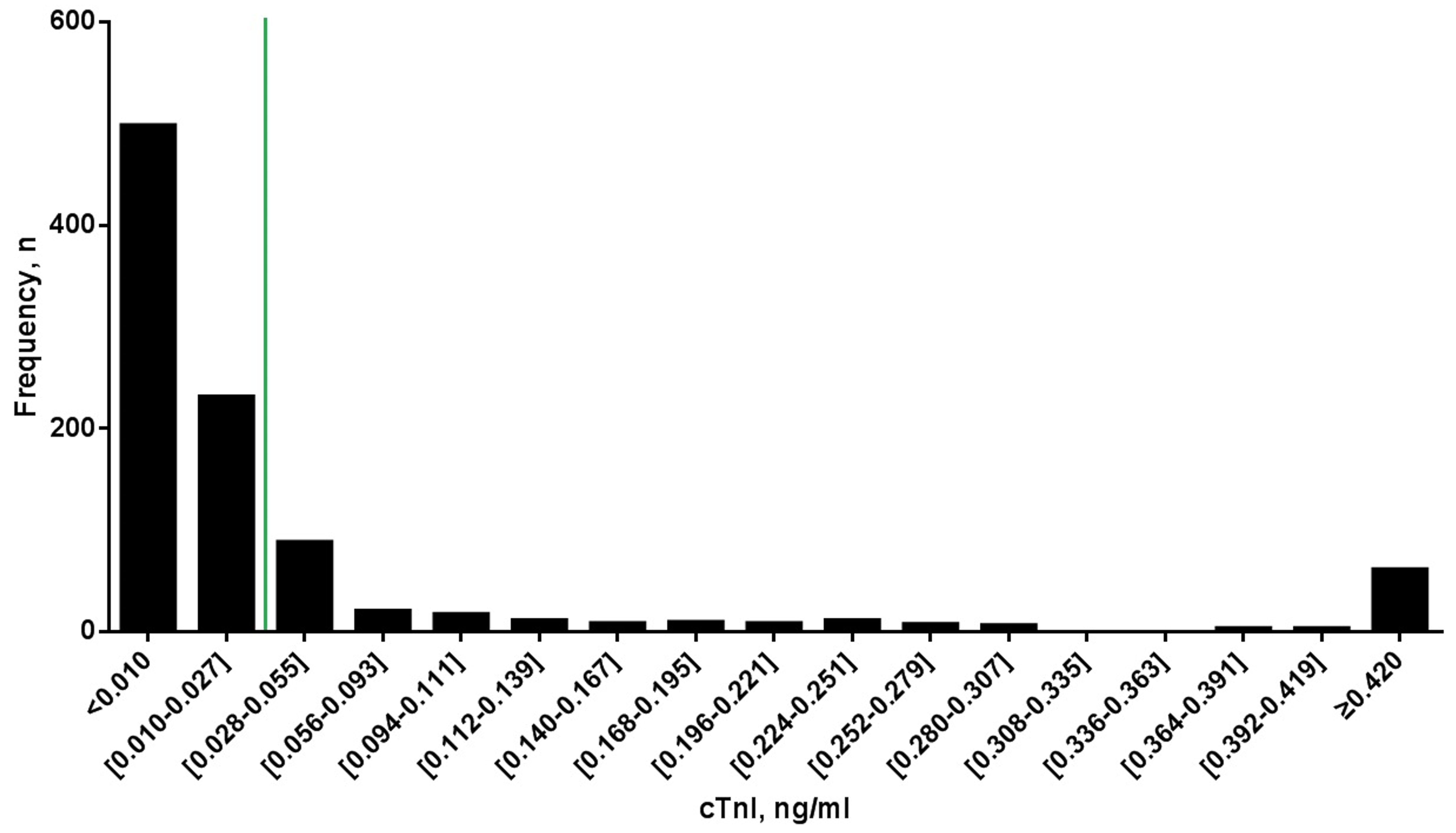

Supplement: Supplementary file 3 — Figure S2. Distribution patterns of peak cTnI levels in ICH patients. Histogram showing the patterns of distribution of peak cardiac troponin I levels in 1004 patients with intracerebral hemorrhage (ICH).N indicates the number of individuals. The green line indicates the 99th percentile of the applied cTnI assay (0.028 ng/mL) in general population. [file CLC-43-338-s003.TIF]
